# Iowa Medical Journal

**Published:** 1869-02

**Authors:** 


### IOWA MEDICAL JOURNAL.

We ask pardon of our subscribers and exchanges for the delay of this, the fourth number of our Medical Journal. Our list of paying subscribers did not justify an earlier issue, yet we should not have discontinued had not ill health prevented this number appearing at the regular time. We now commence the new year with more encouragement and renewed energy, and trust that our professional friends will not let the Journal want for interesting articles, or the means necessary to its future growth and prosperity. Write for it, subscribe for it, pay for it, and ask your friends to do likewise.

